# Development and Validation of a Psychometric Tool for Disability Assessment in Auditory Neuropathy Spectrum Disorder

**DOI:** 10.7759/cureus.98976

**Published:** 2025-12-11

**Authors:** Pradeep Yuvaraj, Aravind Kumar Rajasekaran, Rahina Abubacker, Chethan Kallahalli, Zohra Ghori, Ajith Kumar U

**Affiliations:** 1 Speech Pathology and Audiology, National Institute of Mental Health and Neurosciences, Bengaluru, IND; 2 Audiology, All India Institute of Speech and Hearing, Mysuru, IND

**Keywords:** auditory neuropathy spectrum disorder, disability assessment tool, hearing disability assessment, psychometric validation, questionnaire development and disability certification

## Abstract

Background: Individuals with auditory neuropathy spectrum disorder (ANSD) often demonstrate disproportionately poor speech understanding in noise relative to their pure-tone thresholds. Conventional disability assessments based on pure-tone averages (PTAs) fail to capture these functional limitations, leading to an underestimation of disability. This study aimed to develop and validate the Auditory Neuropathy Perceived Disability Questionnaire (ANPDQ), a condition-specific psychometric tool that assesses functional disability in ANSD.

Method: The study involved 116 individuals aged ≥ 15 years with a confirmed diagnosis of ANSD. The study was conducted in two phases: (1) development and validation of the ANDPQ to assess disability in individuals with ANSD and (2) administration of the questionnaire to evaluate functional disability. The ANPDQ was developed through literature review, patient interviews, and expert input. The final 59-item questionnaire covered functional, psychosocial, and audiological domains.

Results: The ANPDQ demonstrated robust psychometric properties. Eight interpretable components emerged, explaining 74.1% of the total variance. High internal consistency was observed in domains related to speech-in-noise communication, listening effort, and psychological impact. ANPDQ scores showed weak-to-moderate correlations with traditional audiometric measures, highlighting the distinct contribution of self-perceived disability.

Conclusion: The ANPDQ is a valid, reliable tool for assessing functional disability in ANSD. This study proposes a novel composite formula combining PTA and ANPDQ scores to more accurately estimate total hearing disability, for advancing equitable disability certification. The ANPDQ may also serve as a valuable tool for guiding individualized rehabilitation strategies to the unique communicative and psychosocial needs of individuals with ANSD. Grounded in the ICF framework, ANPDQ also holds potential for cross-cultural adaptation and application in global contexts.

## Introduction

Auditory neuropathy spectrum disorder (ANSD) is a unique auditory condition characterized by disordered auditory nerve functioning, typically impaired neural synchrony despite preserved outer hair cell function [1]. Clinically, individuals with ANSD often demonstrate disproportionately poor speech understanding, particularly in noisy environments [1-3]. The prevalence of ANSD varies globally, influenced by diagnostic criteria and population differences. In Western countries, ANSD is primarily reported in infants, with late-onset cases being rare [4,5]. In contrast, Indian studies suggest a higher occurrence of late-onset ANSD, particularly in adolescents and young adults, with prevalence estimates ranging from 0.28% to 5.06% among hearing-impaired population [6-9]. Advancements in diagnostic tools such as otoacoustic emissions (OAEs) and auditory brainstem responses (ABRs) have improved ANSD identification.

Pure-tone audiometry (PTA), the gold standard for hearing threshold evaluation, forms the basis for most hearing disability certification protocols. PTA-based assessment of hearing disability assumes a linear relationship among the degree of hearing loss, speech identification/comprehension, and the associated disability. However, this assumption often does not hold true for individuals with ANSD. Several studies have shown that individuals with ANSD exhibit disproportionately poor speech identification scores relative to their audiometric thresholds [2,10-13]. In some cases, speech perception is severely impaired despite normal or near-normal hearing thresholds [14]. The existing literature indicates that although individuals with ANSD often retain normal loudness perception and sound localization abilities, they demonstrate significant deficits in temporal processing, an essential component for speech understanding, particularly in noisy environments [15-17].

A mismatch between the degree of hearing loss and functional impairment, particularly communicative difficulties in individuals with ANSD, is a critical concern. Many current disability assessment protocols across the globe rely heavily on PTA-based models, often overlooking the functional limitations imposed by conditions like ANSD. While this approach provides a reasonable estimate of disability in individuals with sensory neural hearing loss (SNHL), it is often inadequate for individuals with ANSD. Kim et al. [18] demonstrated the effectiveness of PTA-based criteria for assessing disability in SNHL, owing to the strong relationship between hearing thresholds and speech understanding. However, individuals with ANSD, despite experiencing severe functional communication difficulties, are frequently excluded from disability recognition [19]. This disconnect has substantial implications, as it repudiates the claim for disability certification for persons with ANSD. Their exclusion results in psychological distress, social isolation, and diminished quality of life.

The present study seeks to bridge this gap by developing and validating a questionnaire specifically designed to assess the functional disability experienced by individuals with ANSD. This tool will incorporate both objective audiological data (PTA, ABR, OAEs, speech scores) and subjective dimensions (communication challenges, emotional impact, social participation) in line with the principles of the World Health Organization’s International Classification of Functioning, Disability and Health (ICF) [20]. The questionnaire aims to provide a more accurate and equitable basis for disability determination and supports the larger goal of inclusive policy reform. A validated, ANSD-specific disability tool can also support setting a therapeutic goal to better address the real-life communication challenges in ANSD.

## Materials and methods

Participants

This study was conducted at a tertiary care mental health and neurosciences hospital in southern India between January 28, 2022, and May 31, 2024, and was approved by the Institute Ethics Committee (Approval No.: NIMHANS/30th IEC (BS & NS DIV.)/2021). 

The study sample comprised 116 individuals (females = 69, males = 47) diagnosed with ANSD, with a mean age of approximately 25.8 (8.59) years (range: 15-40 years). The diagnosis of ANSD followed the criteria by Starr et al. [21]: preserved outer hair cell function (indicated by the presence of otoacoustic emissions), absent or markedly abnormal auditory brainstem responses, absent acoustic reflexes, and disproportionately poor speech identification scores relative to pure-tone thresholds. Individuals with a history of neurological disease, head injury, other types of hearing loss, or major medical disorders were excluded. Written informed consent was obtained from all participants.

Given the relatively low prevalence of ANSD, recruiting large samples poses practical constraints. However, the achieved sample size (n = 116), corresponding to a subject-to-item ratio of approximately 2:1, is considered adequate for exploratory factor analysis when communalities are high and factors are well defined. Prior simulation studies support the validity of factor solutions under such conditions [22]. The study was conducted in two phases: (1) development and validation of a questionnaire to assess disability in individuals with ANSD, and (2) administration of the questionnaire to evaluate functional disability. Written informed consent was obtained from all participants prior to data collection.

Phase I: development of the assessment instrument

The first phase involved the development of a psychometric questionnaire encompassing multiple domains reflecting the range of difficulties experienced by individuals with ANSD. The initial version of the ANPDQ was developed and validated in English, reflecting the language commonly used for clinical communication at the tertiary care center where the study was conducted. Although participants hailed from diverse linguistic backgrounds, they were functionally proficient in English. The development process included the following steps.

Literature Review

An extensive review of existing questionnaires and tools used to assess hearing disability and related conditions was conducted. This review helped identify relevant domains, structure, and content of the questionnaire to ensure comprehensive coverage.

Patient Interviews

Semi-structured interviews were conducted with five individuals diagnosed with ANSD who were randomly selected from the database. These interviews explored real-life difficulties encountered in communication, occupational settings, social interactions, and emotional well-being. The responses were used to identify and include items that were not captured by existing instruments.

Focus Group Discussions

A multidisciplinary focus group discussion (FGD) was conducted to refine the preliminary version of the ANPDQ. The group comprised professionals with experience working with individuals with ANSD, including two audiologists, one neurologist, and one clinical psychologist. The FGD followed a semi-structured format and lasted approximately 90 minutes. During the session, each domain and its respective items were reviewed. The group critically evaluated item clarity, conceptual redundancy, cultural relevance, and the comprehensiveness of each domain. The psychologist's input provided valuable insights into the psychosocial framing of disability related questions. This iterative feedback process played a key role in enhancing the face and content validity of the final version of the questionnaire (Supplementary material 1).

Domains

The tool includes both objective audiological indicators and subjective self-report items across nine domains: (1) audiological tests (e.g., PTA, SRT, SIS, SPIN, OAE, ABR, and acoustic reflexes); (2) ease of communication; (3) communication in background noise and reverberation; (4) spatial hearing; (5) quality of heard speech; (6) activities and participation; (7) psychological impact; (8) sensory, motor, and balance issues; and (9) listening effort and fatigue. Each item was rated on a 5-point Likert-type scale, ranging from "Never" to "Every time," capturing the frequency of real-world difficulties experienced by individuals with ANSD.

Content Validation

The expert validation was undertaken in two sequential phases to ensure the relevance, clarity, and appropriateness of the domains and individual items.

In the first phase, the preliminary version of the questionnaire was reviewed by five professionals with extensive clinical and research experience in the field of ANSD. These experts evaluated both the proposed domains and the individual questions within each domain. A structured rating format was used, wherein each domain and item was assessed on a 5-point Likert scale, ranging from 1 (Strongly Disagree) to 5 (Strongly Agree). The goal was to obtain consensus on the content validity of each component. Only items and domains that received a mean score of greater than 4 across the five experts were retained for inclusion in the revised version of the instrument. Items scoring below this threshold were modified either based on expert suggestions or eliminated to improve the overall content validity and focus of the tool.

In the second phase, the revised questionnaire was circulated to a different set of five experts, all of whom were also actively engaged in clinical and/or academic work related to ANSD. This step served as the final validation. The experts again reviewed the content for conceptual clarity, domain appropriateness, item redundancy, and overall comprehensiveness. Feedback from this phase was used to make minor refinements and confirm the final structure of the questionnaire. This two-tiered expert validation process was to ensure that the instrument was both theoretically grounded and clinically relevant, enhancing its utility for assessing functional disability in individuals with ANSD.

The questionnaire was pilot-tested on a small sample of five individuals diagnosed with ANSD to assess its face validity. These participants were asked to complete the questionnaire and provide feedback on the clarity, relevance, and comprehensiveness of the items. The pilot testing helped identify any ambiguous or confusing questions and ensured that the content was appropriate and understandable. Based on the feedback received, minor revisions were made to improve the wording and format of the questionnaire.

Phase II: questionnaire administration

The validated questionnaire was administered to all participants with a confirmed diagnosis of ANSD. Participants completed the questionnaire either in person under the supervision of trained research staff. The questionnaire was designed to capture both objective audiological data and subjective self-perceptions related to hearing difficulties and their impact on daily life. Clear instructions were provided to ensure accurate and complete responses, and data collection was conducted in a standardized manner to maintain consistency across all participants.

All statistical analyses were conducted using IBM SPSS Statistics for Windows, Version 22.0 (Released 2013; IBM Corp., Armonk, NY, USA). Descriptive statistics were computed for demographic and audiological variables. To examine the construct validity of the ANPDQ, factor analysis was performed using the principal component analysis (PCA) with promax rotation. Internal consistency of the scale and subdomains was evaluated using Cronbach’s alpha, with thresholds of ≥0.70 denoting acceptable reliability. Test-retest reliability was assessed in a subsample of 26 participants using Pearson’s correlation coefficient, intraclass correlation coefficient (ICC), and Bland-Altman plots. Standard error of measurement (SEM) and minimal detectable change (MDC) were also calculated to assess measurement precision and clinical utility.

## Results

The ANPDQ was administered to a total of 116 individuals with auditory neuropathy. The sample consisted of 59.5% females (n = 69) and 40.5% males (n = 47), with a male-to-female ratio of approximately 1:1.47. The mean age of illness onset was 16.79 (6.00) years, and the average duration of illness was 9.06 (7.75) years. Table 1 presents the mean and standard deviation for each item in the questionnaire. Within each section of the ANPDQ, the questions are arranged in descending order of average scores. The distribution of scores across the subsections of the ANPDQ followed the expected pattern. Participants reported the greatest difficulty in the "communication in background noise and reverberation" subsection, followed by the "ease of communication" subsection, among others.

**Table 1:** Mean and one standard deviation for ANPDQ ANPDQ: Auditory Neuropathy Perceived Disability Questionnaire.

Domains and questions	Mean	Standard deviation
Ease of communication	0.63	0.2
1. Do you have difficulty conversing with your family members?	2.16	1.09
2. Do you easily understand the speech of an unfamiliar person?	2.58	1.10
3. Do you have difficulty understanding the speech of others in one-to-one conversation?	2.03	1.15
4. Do you ask for repetition during conversation?	2.69	1.11
5. Do you take longer to respond to a question?	2.29	1.19
6. Do you hesitate to initiate conversation?	2.37	1.29
7. Do you easily converse over the telephone?	2.88	1.33
8. Do you have difficulty understanding speech without looking at the speaker?	2.84	1.08
9. Do you easily follow conversation even without knowing the context?	2.67	1.17
10. Do you miss out on a portion of the speech in the conversation?	2.80	1.01
Communication in the background noise and reverberation	0.73	0.17
11. Do you easily carry out conversation in a crowded place (e.g., market)?	3.34	1.14
12. Do you have difficulty listening to the TV in the background noise (e.g., family members talking, AC/fan running)?	3.22	1.04
13. Do you have difficulty carrying out a telephonic conversation in the background noise (e.g., busy street /traffic)?	3.47	0.82
14. Do you easily understand speech in a classroom/theater/open hall/lecture room?	2.61	1.11
15. Do you misunderstand parts of the conversation in the background noise?	3.10	0.96
16. Do you have difficulty carrying out conversation with your fellow passengers during travel (e.g., auto, bus, train, flight)?	3.31	0.99
17. Do you easily carry out one-to-one conversation in a quiet place?	1.60	1.24
Spatial hearing	0.53	0.26
18. Do you have difficulty judging the direction of the vehicle by listening?	2.04	1.33
19. Do you have difficulty determining the source of the sound by listening?	2.16	1.17
20. Do you have difficulty judging the distance of a sound source by listening?	2.24	1.23
21. Do you have difficulty recognizing who is talking in a crowd?	2.18	1.38
Quality of heard speech and non-speech sounds	0.45	0.22
22. Do you find it easy to recognize people by their voices?	1.77	1.46
23. Do you feel clarity of speech is poor over the telephone?	3.23	1.04
24. Do you feel environmental sounds sound natural to you?	1.39	1.30
25. Do you have difficulty perceiving the emotion/intention of the talker by the voice?	1.84	1.34
26. Do you find clarity of speech is better when spoken louder?	2.32	1.40
27. Do you have difficulty differentiating between speech sounds and environmental sounds?	1.70	1.43
28. Do you have difficulty appreciating the quality of music?	2.28	1.28
29. Do you have difficulty identifying the non-speech sounds?	1.59	1.39
Activities and participation	0.54	0.24
30. Do you have difficulty attending school/college/work due to hearing issues?	2.60	1.20
31. Do you have difficulty attending social gatherings?	2.57	1.26
32. Do you find it easy to socialize with others?	2.44	1.23
33. Do you have difficulty involving yourself in recreational and leisure activities?	1.74	1.36
34. Do you have difficulty maintaining friendly relationships?	1.75	1.34
35. Do you find it difficult to acquire new skills due to hearing issues?	2.03	1.27
Psychological	0.44	0.21
36. Do you feel sad because of hearing difficulty?	2.59	1.15
37. Have you lost interest in other people and activities?	2.13	1.25
38. Do you feel frustrated because of hearing difficulty?	2.33	1.14
39. Do you have a fear of failure because of hearing difficulty?	2.15	1.32
40. Do you feel worthless because of hearing difficulty?	1.73	1.34
41. Do you feel you have adequate sleep?	0.73	1.14
42. Do you feel your eating pattern is disturbed?	0.44	0.93
43. Do you feel anxious because of hearing difficulty?	2.23	1.21
44. Do you feel irritable because of hearing difficulty?	2.16	1.12
45. Do you feel uncertain about your future because of hearing difficulty?	2.04	1.37
46. Do you feel neglected because of hearing difficulty?	1.86	1.36
47. Do you have anger issues because of hearing difficulty?	2.00	1.32
48. Do you feel emotionally drained due to hearing difficulty?	1.89	1.31
49. Do you have memory issues?	0.86	1.19
Sensory, motor, and balance	0.32	0.2
50. Do you experience giddiness?	0.91	1.04
51. Do you have difficulty maintaining balance in dark/eyes closed conditions?	1.78	1.38
52. Do you easily climb stairs?	1.37	1.50
53. Do you hear ringing sound in your ears?	1.64	1.40
54. Do you have difficulty standing or walking?	1.42	1.35
55. Do you find it easy to perform everyday tasks independently?	0.55	1.07
Listening effort and fatigue	0.57	0.22
56. Do you have to concentrate more during conversation?	2.87	1.10
57. Do you get fatigued easily due to listening?	1.68	1.19
58. Do you feel exhausted after a conversation?	1.58	1.25
59. Do you think you can listen effortlessly over the telephone?	3.09	1.31
Overall disability	0.52	0.16

**Figure 1:**
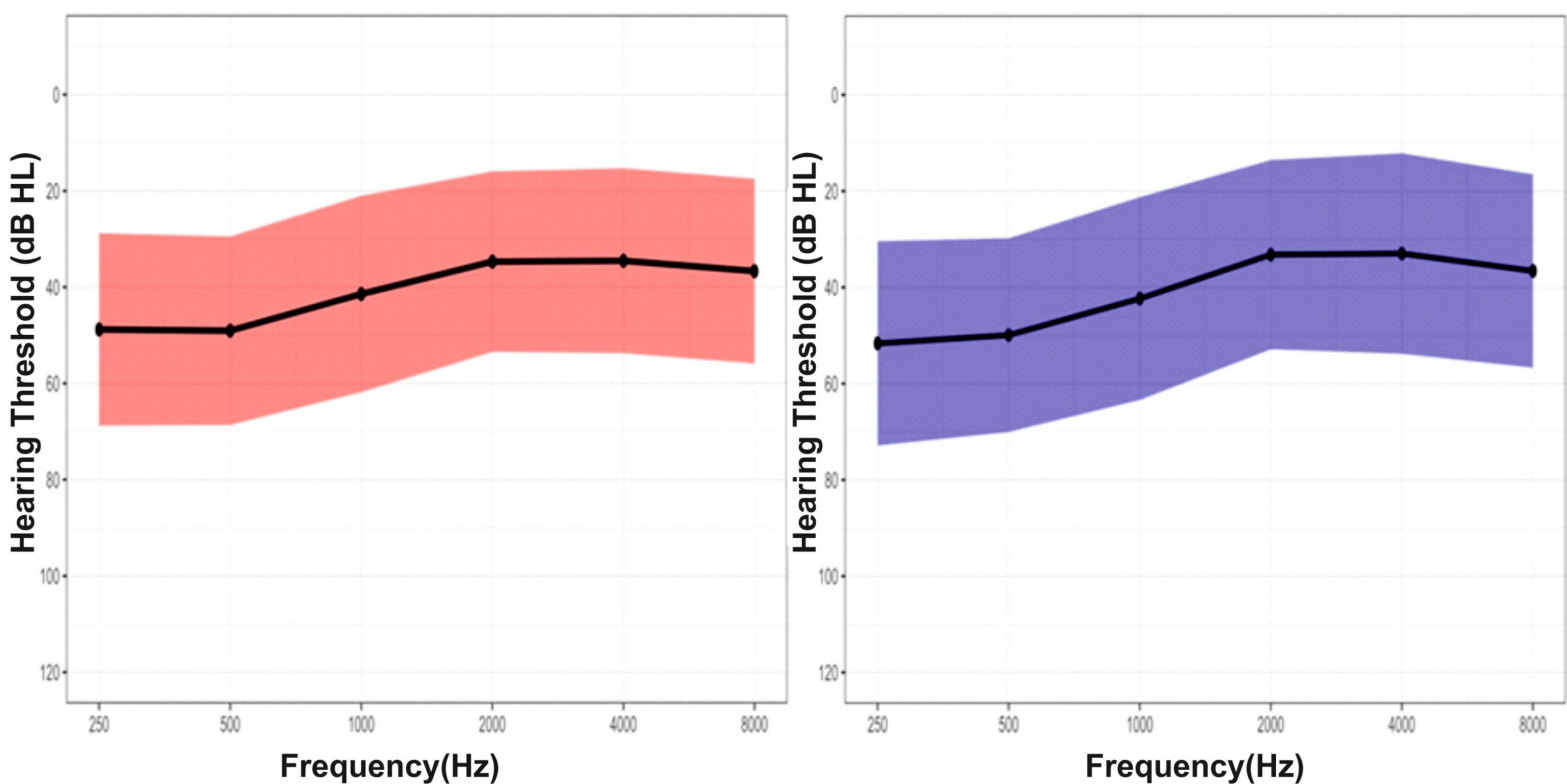
Mean audiometric threshold for the right and left ears Solid black lines represent average hearing thresholds (dB HL), and shaded regions indicate one standard deviation.

Figure 1 illustrates the mean and one standard deviation of hearing thresholds in the right and left ears across octave frequencies ranging from 250 Hz to 8 kHz.

**Figure 2:**
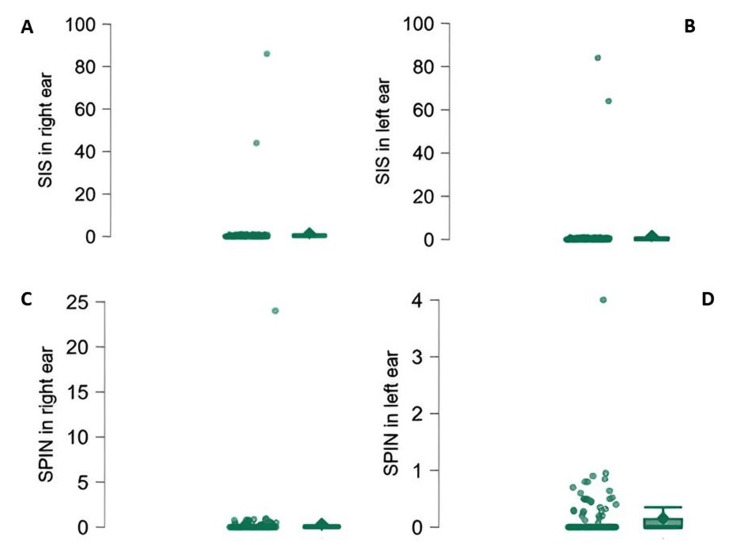
Distribution of speech identification scores (SIS; top panels) and speech perception in noise (SPIN; bottom panels) for the right (left column) and left (right column) ears Each dot represents an individual data point.

Figures 2a-2d depict the speech identification scores in quiet and at a 0 dB signal-to-noise ratio (SNR). Most individuals had no measurable speech identification scores in quiet, and this number further increased at 0 dB SNR. These results indicate that speech understanding is disproportionately poor relative to pure-tone hearing thresholds in individuals with auditory neuropathy.

To explore the underlying structure of the 59-item questionnaire, PCA was conducted. Prior to performing PCA, the suitability of the data for factor analysis was assessed using the Kaiser-Meyer-Olkin (KMO) test of sampling adequacy and Bartlett’s test of sphericity, within an exploratory factor analysis framework with promax rotation. The KMO measure indicated that the data were adequate for factor analysis, with all values exceeding the recommended threshold of 0.50 [23], except for all items in the sensory-motor balance subsection. As a result, items from this subsection were excluded from the PCA. Bartlett’s test of sphericity was significant (p < 0.001), indicating that the data were suitable for factor analysis. Components with eigenvalues greater than 1 were retained, and visual inspection of the scree plot supported the retention of eight components. These eight components cumulatively accounted for 74.1% of the total variance. The rotated component matrix revealed clear and interpretable factor loadings, with all items loading ≥ 0.40 on a single component. Table 2 presents the rotated component loadings, which ranged from 0.40 to 0.98, suggesting that the components adequately represented the underlying items. Supplementary material 2 reorganizes the ANPDQ based on the PCA results.

**Table 2:** Results of the PCA PCA: principal component analysis.

	RC1	RC2	RC3	RC4	RC5	RC6	RC7	RC8	RC9	RC10
Q47	0.967	-	-	-	-	-	-	-	-	-
Q44	0.887	-	-	-	-	-	-	-	-	-
Q38	0.804	-	-	-	-	-	-	-	-	-
Q48	0.741	-	-	-	-	-	-	-	-	-
Q36	0.650	-	-	-	-	-	-	-	-	-
Q11	-	1.026	-	-	-	-	-	-	-	-
Q59	-	0.895	-	-	-	-	-	-	-	-
Q7	-	0.788	-	-	-	-	-	-	-	-
Q14	-	0.762	-		-	-	-	-	-	-
Q9	-	0.507	-	-	-	-	-	-	-	-
Q2	-	0.438	-	-	-	-	-	-	-	-
Q19	-	-	0.847	--	-	-	-	-	-	-
Q18	-	-	0.785	-	-	-	-	-	-	-
Q29	-	-	0.654	0.452	-	-	- -	-	-	-
Q25	-	-	0.508	0.458	-	-	-	-	-	-
Q28	-	-	0.503	-	-	-	-	-	-	-
Q27	-	-	0.493	0.599	-	-	-	-	-	-
Q5	-	-	0.469	-	-	-	-	-	-	-
Q20	-	-	0.428	-	-	-	-	-	-	-
Q3	-	-	-	0.762	-	-	-	-	-	-
Q33	-	-	-	0.651	-	-	-	-	-	-
Q34	-	-	-	0.614	-	-	-	-	-	-
Q35	-	-	-	0.469	-	-	-	-	-	-
Q21	-	-	-	-	0.697	-	-	-	-	-
Q30	-	-	-	-	0.647	-	-	-	-	-
Q40	-	-	-	-	0.583	-	-	-	-	-
Q37	-	-	-	-	0.531	-	-	-	-	-
Q22	-	-	-	-	0.486	-	-	-	0.561	-
Q31	-	-	-	-	0.463	-	-	-	-	-
Q45	-	-	-	-	0.462	-	-	-	-	-
Q46	-	-	-	-	0.407	0.478	-	-	-	-
Q57	-	-	-	-	-	0.856	-	-	-	-
Q58	-	-	-	-	-	0.762	-	-	-	-
Q16	-	-	-	-	-	-	0.933	-	-	-
Q15	-	-	-	-	-	-	0.834	-	-	-
Q13	-	-	-	-	-	-	0.521	-	-	-
Q12	-	-	-	-	-	-	0.475	-	-	-
Q4	-	-	-	-	-	-	-	0.980	-	-
Q6	-	-	-	-	-	-	-	0.733	-	-
Q24	-	-	-	-	-	-	-	-	0.726	-
Q10	-	-	-	-	-	-	-	-	0.589	-
Q49	-	-	-	-	-	-	-	-	-	0.795
Q1	-	-	-	-	-	-	-	-	-	-

The PCA findings largely aligned with the original categorization of the questionnaire items into subsections and may be used to assess the disability in individuals with ANSD.

Component 1 consisted exclusively of five items from the psychological subsection. Component 2 comprised items primarily from the background noise and reverberation subsection, with the exception of Q59. Component 3 included items from the spatial and quality of heard speech subsection. Notably, Q5 ("Do you take a longer time to respond?") showed a higher loading on this component. Component 4 was primarily composed of items from the activity and participation subsection, except for Q21. Component 5 included items from the psychological subsection, particularly those addressing the impact of hearing difficulties on mental well-being. While Q30 and Q31 originated from the activity and participation subsection, their content also reflects emotional responses to hearing difficulties: Q30 - “Do you have difficulty attending school/college/work due to hearing issues?” Q31 - “Do you have difficulty attending social gatherings?” Component 6 consisted of items related to listening effort, and Component 7 included items addressing speech understanding in difficult listening situations. Although the conceptual focus of these components overlapped, PCA differentiated them into distinct groups. Components 8-10 included items related to clarity of heard speech and audibility.

Items Q1, Q8, Q23, Q29, Q32, Q43, and Q56 did not load strongly on any component, indicating weaker associations with the identified factors.

**Figure 3:**
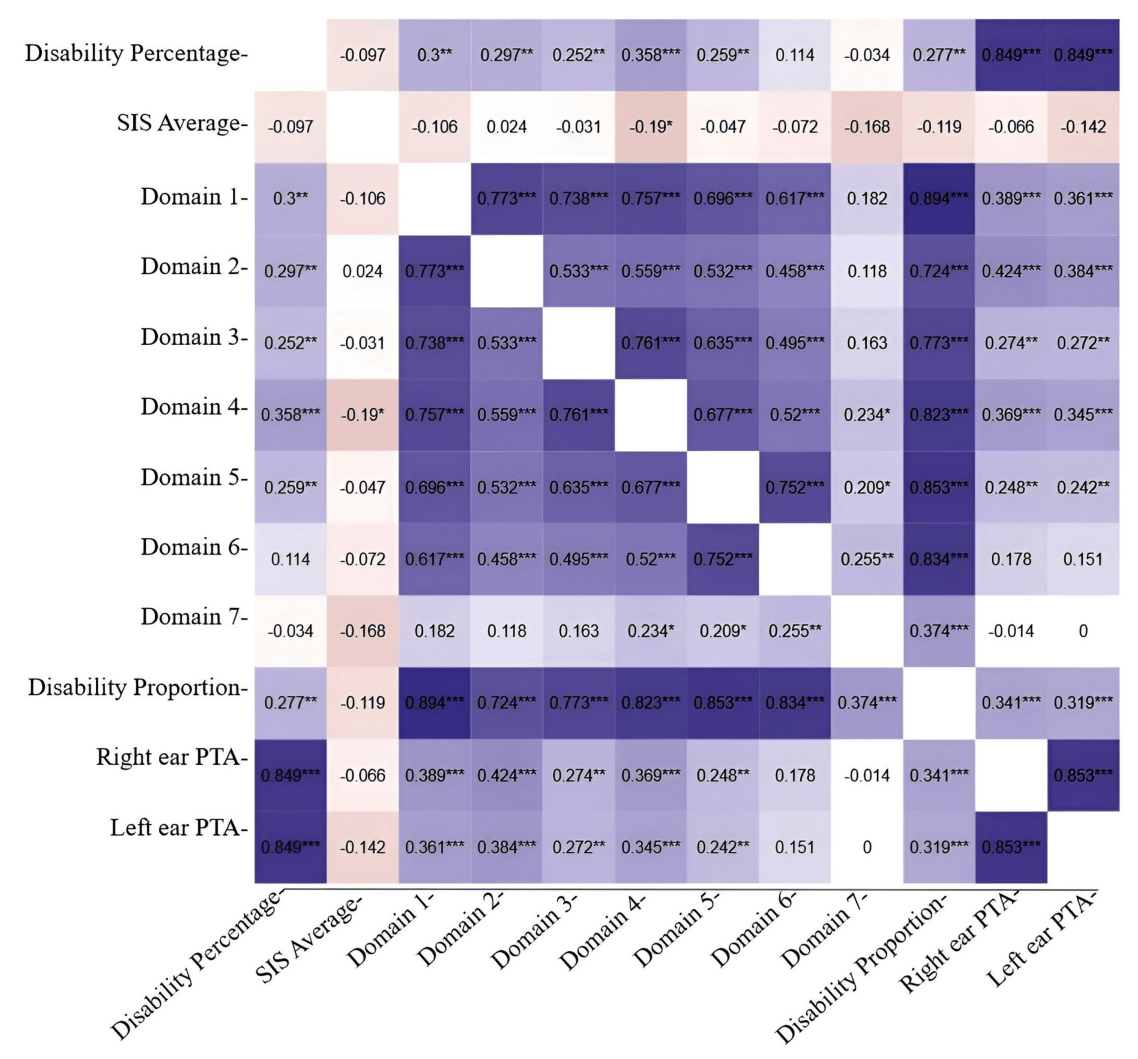
Heatmap representing the correlation among the variables Color intensity represents the strength of the correlation, with darker shades indicating stronger relationships. Domains: 1, ease of communication; 2, communication in the background noise and reverberation; 3, spatial hearing; 4, quality of heard speech and non-speech sounds; 5, activities and participation; 6, psychological impact; 7, sensory, motor, and balance. PTA: pure-tone audiometry, SIS: speech identification scores. *Significant.

Figure 3 shows the correlation heatmap between the ANPDQ scores, subsection-wise, and different other hearing metrics, such as the mean speech identification scores of the right and left ear and the percentage of hearing disability. As can be seen, the ANPDQ overall and subsection-wise scores had weak-to-moderate correlations with the percentage disability. Speech identification scores in quiet did not correlate with the overall or any of the subsections of the ANPDQ questionnaire.

Internal consistency of the ANPDQ

The internal consistency of the ANPDQ was evaluated using Cronbach’s alpha. Analysis was conducted on all 59 items of the questionnaire across valid cases (N = 116). The overall Cronbach’s alpha for the full scale was 0.960, indicating excellent internal consistency. When standardized items were considered, the alpha slightly increased to 0.961, further supporting the homogeneity of the scale. Each item’s contribution to the total scale reliability was examined. The “Cronbach's Alpha if Item Deleted” values ranged between 0.959 and 0.961, suggesting that removal of any individual item would not significantly improve or reduce the overall reliability of the scale. Corrected item-total correlations for most items were moderate to high, indicating that items are sufficiently correlated with the overall scale and contribute meaningfully to the construct being measured (Table 3).

**Table 3:** Internal consistency of the ANPDQ (Cronbach’s alpha) ANPDQ: Auditory Neuropathy Perceived Disability Questionnaire.

Domains	Questions	Cronbach's Alpha
Ease of communication	Q1	0.959
	Q2	0.960
	Q3	0.959
	Q4	0.960
	Q5	0.959
	Q6	0.959
	Q7	0.959
	Q8	0.959
	Q9	0.959
	Q10	0.959
Communication in the background noise and reverberation	Q11	0.960
	Q12	0.960
	Q13	0.959
	Q14	0.960
	Q15	0.960
	Q16	0.960
	Q17	0.960
Spatial hearing	Q18	0.959
	Q19	0.959
	Q20	0.959
	Q21	0.959
Quality of heard speech and non-speech sounds	Q22	0.960
	Q23	0.959
	Q24	0.960
	Q25	0.959
	Q26	0.961
	Q27	0.959
	Q28	0.959
	Q29	0.959
Activities and participation	Q30	0.959
	Q31	0.959
	Q32	0.959
	Q33	0.959
	Q34	0.959
	Q35	0.959
Psychological	Q36	0.959
	Q37	0.959
	Q38	0.960
	Q39	0.959
	Q40	0.959
	Q41	0.961
	Q42	0.960
	Q43	0.959
	Q44	0.959
	Q45	0.959
	Q46	0.959
	Q47	0.960
	Q48	0.959
	Q49	0.960
Sensory, motor, and balance	Q50	0.960
	Q51	0.961
	Q52	0.961
	Q53	0.960
	Q54	0.961
	Q55	0.961
Listening effort and fatigue	Q56	0.959
	Q57	0.960
	Q58	0.959
	Q59	0.960

Test-retest reliability

**Figure 4:**
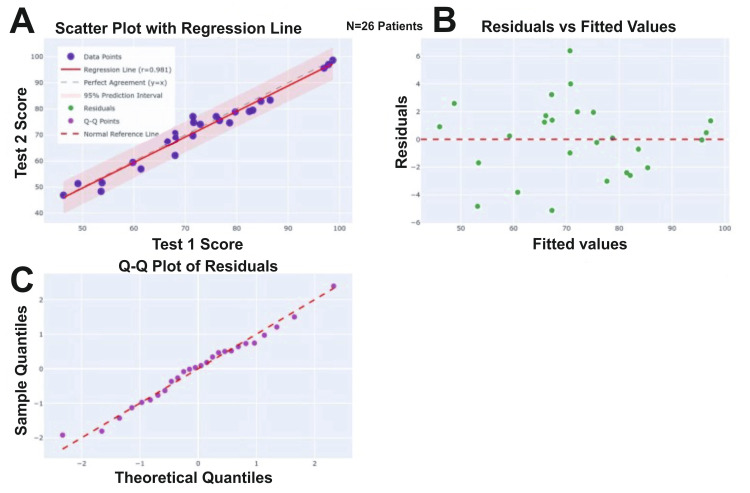
Test-retest reliability of ANPDQ using Pearson correlation analysis (A) Scatter plot with regression line showing a strong positive correlation between Test 1 and Test 2 scores. The red dashed line represents the regression fit with the shaded band indicating the 95% prediction interval, while the red dashed line denotes the line of perfect agreement. (B) Residuals versus fitted values plot confirming random distribution of residuals around zero, indicating good model fit and absence of heteroscedasticity. (C) Q-Q plot of residuals illustrating that residuals follow a normal distribution, as data points closely align with the reference line. Overall, the analysis indicates excellent test-retest reliability and model validity. ANPDQ: Auditory Neuropathy Perceived Disability Questionnaire.

**Figure 5:**
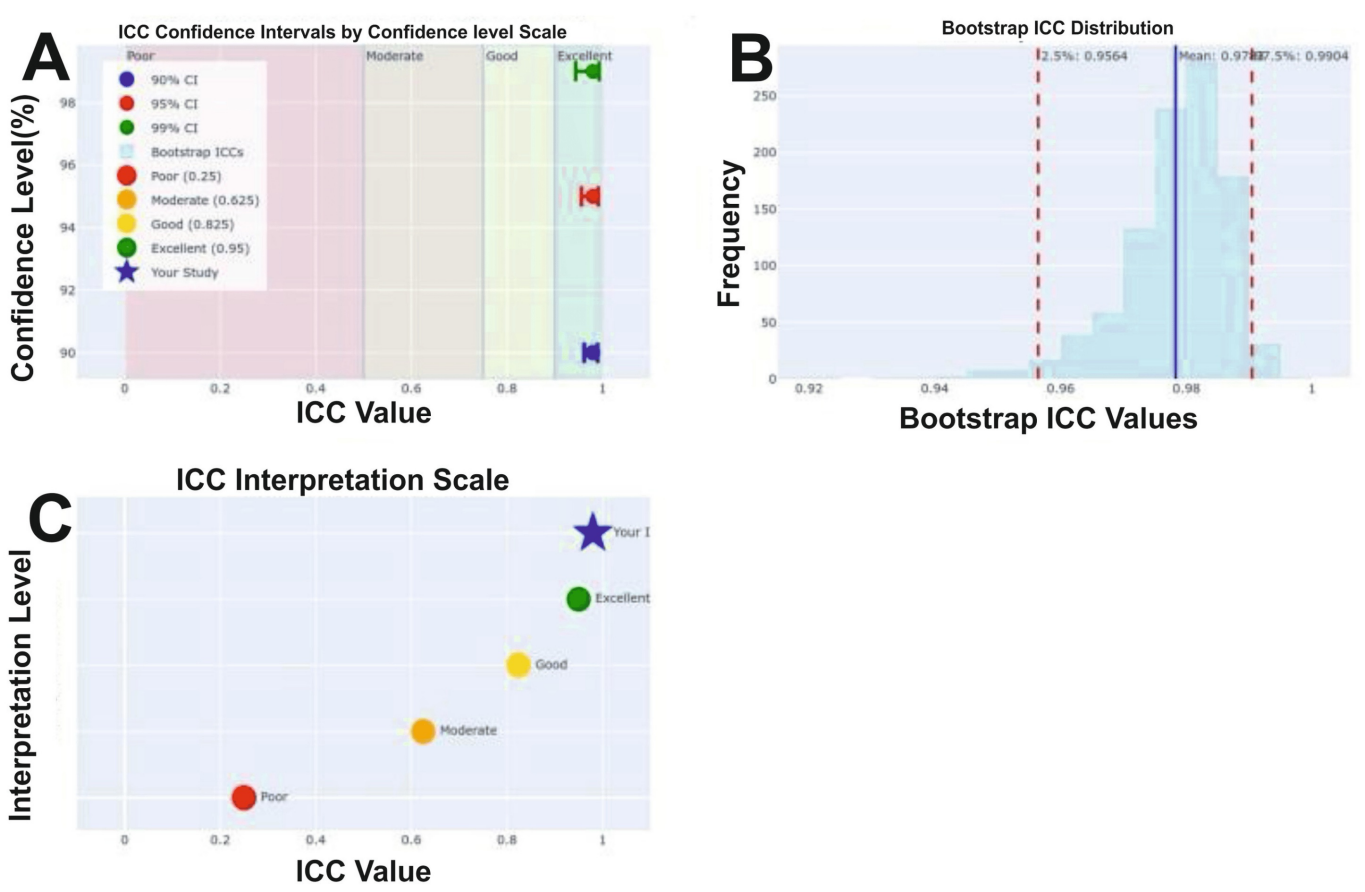
Intraclass correlation coefficient (ICC) analysis for test-retest reliability of ANPDQ (A) ICC confidence intervals across 90%, 95%, and 99% levels, with classification zones for poor, moderate, good, and excellent reliability. The study’s ICC (blue star) is within the “excellent” range (>0.9). (B) Bootstrap ICC distribution (10,000 resamples), showing a narrow, high-reliability range. (C) ICC interpretation scale, placing the study’s ICC well within the excellent category. ANPDQ: Auditory Neuropathy Perceived Disability Questionnaire.

The test-retest reliability was assessed over a two-week interval in a subsample of 26 patients. The results demonstrated excellent reliability, with a Pearson correlation coefficient (r = 0.981) and an intraclass correlation coefficient (ICC = 0.980) both exceeding the accepted threshold of 0.90 for excellent reliability (Figures 4, 5; Tables 4, 5). 

**Table 4:** Pearson correlation statistics This table summarizes correlation statistics, including a Pearson correlation coefficient, with a highly significant p-value.

Statistic	Value	Interpretation
Pearson correlation (r)	0.9811	(0.9577, 0.9916)
R-squared (r²)	0.9629	96.3% variance explained
p-value	1.24e-18	Highly significant
Sample size	26	-
Regression equation	Y = 0.980x + 0.620	-
Standard error	0.0394	-
Residual standard deviation	2.668	-
Mean absolute error	2.115	-

**Table 5:** Intraclass correlation coefficient (ICC) analysis

Statistic	Value	Notes
ICC (3,1)	0.9800	Excellent
95% CI (F-distribution)	0.9562, 0.9909	Width: 0.0347
90% CI	0.9615, 0.9896	Width: 0.0282
99% CI	0.9435, 0.9930	Width: 0.0494
Bootstrap mean	0.9783	SC: 0.0089
Bootstrap 95% CI	0.9564, 0.9904	Percentile method
Sample size	26	Patients
Interpretation	Excellent	ICC > 0.90

**Figure 6:**
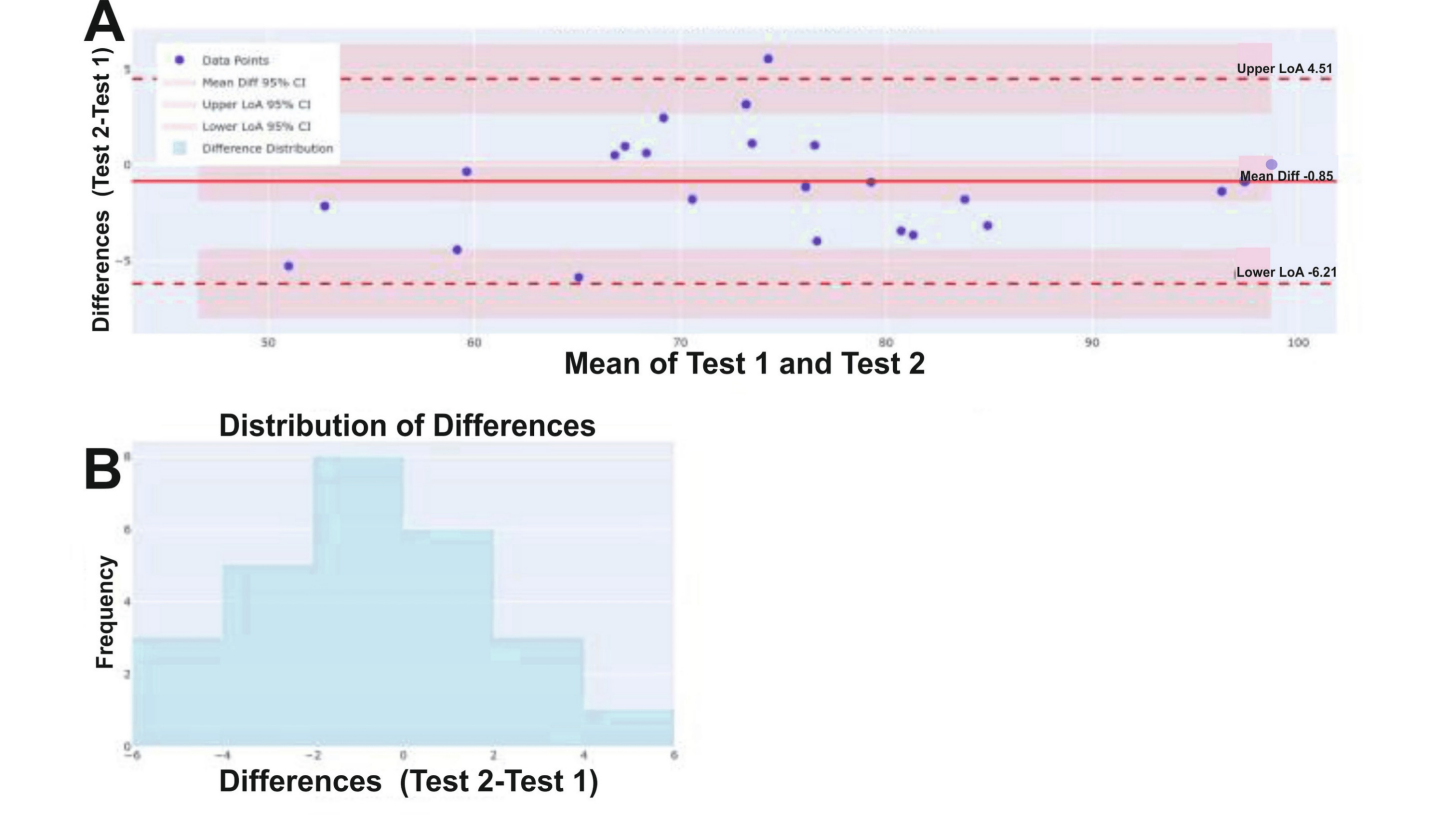
Bland-Altman analysis for test-retest agreement of ANPDQ scores (A) Solid central red line indicates the mean difference, while the dashed red lines represent the upper and lower limits of agreement. (B) Distribution of score differences, illustrating a normal spread around the mean. ANPDQ: Auditory Neuropathy Perceived Disability Questionnaire.

The SEM was low at 1.93, indicating high precision, and the MDC was calculated as 5.36, serving as a threshold for clinically meaningful change. Furthermore, Bland-Altman analysis (Figure 6; Table 6) revealed minimal systematic bias and acceptable limits of agreement, confirming the consistency of the instrument across repeated administrations (Tables 7, 8).

**Table 6:** Bland-Altman analysis: test-retest agreement

Statistic	Value	95% CI
Mean difference	-0.849	-1.90, 0.203
SD of differences	2.736	-
Upper limit of agreement	4.513	2.691, 6.334
Lower limit of agreement	-6.211	-8.032, -4.389
Range of agreement	10.724	-
% within LoA	96.2%	-

**Table 7:** Test-retest scores, Pearson correlation (r), and intraclass correlation coefficient (ICC)

Patient ID	Test 1	Test 2	Difference	Pearson r (excluded)	ICC (excluded)
P01	82.45	79	-3.45	0.982	0.92
P02	72.93	74.05	1.13	0.982	0.70
P03	84.72	82.91	-1.8	0.981	0.99
P04	97.85	96.97	-0.88	0.978	1.00
P05	71.49	69.68	-1.81	0.981	0.64
P06	71.49	77.04	5.56	0.985	0.20
P07	98.69	98.65	-0.04	0.978	1.00
P08	86.51	83.34	-3.17	0.981	0.97
P09	67.96	70.43	2.47	0.982	0.76
P10	83.14	79.48	-3.66	0.982	0.92
P11	68.05	68.68	0.63	0.981	0.99
P12	68.01	62.14	-5.88	0.984	0.75
P13	78.63	74.64	-3.98	0.982	0.71
P14	46.3	46.89	0.59	0.978	1.00
P15	49.13	51.34	2.22	0.980	1.00
P16	66.57	67.08	0.51	0.981	1.00
P17	59.81	59.46	-0.35	0.980	1.00
P18	79.71	78.81	-0.9	0.981	0.99
P19	61.38	56.94	-4.44	0.982	0.95
P20	53.82	51.66	-2.16	0.980	0.99
P21	96.98	95.6	-1.38	0.979	1.00
P22	71.61	74.78	3.17	0.983	0.14
P23	76.01	77.04	1.03	0.981	0.97
P24	53.63	48.34	-5.29	0.982	0.97
P25	66.83	67.81	0.97	0.981	0.98
P26	76.66	75.51	-1.16	0.981	0.96

**Table 8:** Summary of test-retest reliability and agreement metrics for ANPDQ ANPDQ: Auditory Neuropathy Perceived Disability Questionnaire.

Statistic	Value	Interpretation
Pearson correlation (r)	0.981	Excellent (≥0.90)
ICC (3,1)	0.98	Excellent (≥0.90)
Standard error of measurement	1.93	Good precision
Minimal detectable change (95%)	5.36	Acceptable range
Mean difference	-0.85	Minimal bias
SD of differences	2.74	Low variability
Lower limit of agreement	-6.21	95% agreement
Upper limit of agreement	4.51	95% agreement
Coefficient of variation (%)	19.47	Acceptable variation
Systematic bias (p-value)	0.126	No systematic bias

## Discussion

Despite experiencing severe communication deficits, the functional and psychosocial impact of ANSD is overlooked in clinical and disability frameworks. This is largely due to the condition's diverse clinical manifestations, which complicate standard assessment methods. The present study aims to develop a novel, condition-specific approach for evaluating disability in ANSD using a psychometric questionnaire, which was designed and administered to 116 participants.

The PCA conducted in this study provides robust empirical support for the structural validity of the ANPDQ. The emergence of eight distinct components, cumulatively accounting for over 74% of the variance, indicates that the questionnaire comprehensively captures the multifaceted nature of disability experienced by individuals with ANSD. Importantly, the factor structure closely aligned with the original domain classification, offering both construct validity and clinical relevance.

Among the subsections, the "communication in background noise and reverberation" and "psychological impact" subsections showed the highest item loadings and internal consistency. These domains are particularly significant in ANSD, where neural desynchrony severely affects temporal resolution and speech perception in complex auditory environments [15,16,24]. The strong representation of these dimensions in the PCA supports the validity of the ANPDQ; that is, it reflects the real-world challenges encountered by affected individuals. Moderate-to-weak correlations between the ANPDQ scores and traditional audiometric measures such as PTA, SIS, and percent disability highlight the discordance between clinical thresholds and perceived disability in ANSD. These findings are consistent with prior studies demonstrating that individuals with ANSD often present with near-normal PTA yet face severe communicative impairments [10,13]. Notably, the psychological and participation-related components showed minimal correlation with audiometric metrics, further reinforcing that auditory thresholds do not adequately capture the psychosocial and functional burdens of the disorder.

This disconnect underscores a central argument of this study: that questionnaire-based assessment is not merely supplementary, but essential in quantifying disability in ANSD. While tools like the Hearing Handicap Inventory for Adults (HHIA) or Speech, Spatial and Qualities of Hearing Scale (SSQ) have demonstrated the broader utility of subjective assessments [25], we did not include a comparison with existing hearing disability or auditory processing disorder (APD) questionnaires in this study, as these tools are typically designed for individuals with sensorineural hearing loss or central auditory processing issues. Such instruments may not fully reflect the specific challenges experienced by this population. Therefore, we focused on developing a condition-specific tool rather than benchmarking it against less-aligned measures. The ANPDQ addresses this gap by including ANSD-specific domains that are rarely captured in general-purpose scales. Future work may examine how the ANPDQ compares with broader auditory disability scales across different clinical populations.

Furthermore, the PCA revealed that certain items particularly those concerning participation, listening effort, and speech understanding under complex auditory conditions formed distinct components, suggesting that individuals perceive and categorize these challenges as discrete aspects of their experience. This nuanced structure enhances the interpretability and clinical utility of the tool. For example, clinicians can now pinpoint whether a person’s primary difficulty lies in cognitive effort, auditory localization, or psychosocial participation, allowing for more targeted rehabilitation strategies.

These findings may have implications for disability assessment and rehabilitation frameworks globally. In many countries, hearing disability certification still relies primarily on PTA, which may not adequately reflect the communicative or psychosocial burden of conditions like ANSD [26]. While the Indian disability legislation [27] was referenced as one example, the issue it illustrates (under-recognition of functional disability in individuals with preserved audiometric thresholds) is relevant worldwide. The ANPDQ, developed using internationally accepted scale development protocols and grounded in the WHO’s ICF framework, offers a condition-specific approach that can complement existing assessment systems and inform both clinical care and disability classification. As Shetty et al. [19] rightly argue, disability certification models must evolve to accommodate condition-specific criteria. The current model inadvertently excludes a significant group of individuals from accessing governmental support, despite their real-world challenges being often more severe than those with conventional sensorineural hearing loss. The ANPDQ thus serves as a policy-relevant tool that could pave the way for more equitable and individualized certification protocols.

The test-retest interval of 15-20 days used in this study is consistent with recommended guidelines for assessing temporal stability of patient-reported outcome measures. According to Park et al. [28], such intervals are appropriate as they are sufficiently long to reduce recall bias, yet short enough to minimize the likelihood of real clinical change, particularly in conditions like ANSD, where functional status remains relatively stable over short periods.

To bridge the gap between objective measures and lived disability, this study introduces a model that integrates PTA-based percentages with ANPDQ-derived functional disability scores. The proposed formula incorporates a headroom factor to avoid double counting and ensures that subjective experiences are weighted more heavily when PTA underestimates impairment, as is often the case in ANSD. This hybrid model can support more accurate functional assessments in clinical and rehabilitative contexts and may be adapted for use within various national or institutional disability determination systems.

Proposed model for disability estimation in ANSD

We propose a model for estimating hearing disability in individuals with ANSD that integrates both objective (PTA-based) and subjective (ANPDQ-based) indicators into a unified percentage, though this formula is exploratory and requires further validation.

The proposed formula

\(Disability % = PTA-based % + [ k × ANPDQ % × (1 - PTA-based % / 100) ]\

The above formula provides an estimation of the total hearing disability by combining (i) PTA-based disability percentage, (ii) functional disability percentage derived from the ANPDQ, and (iii) a headroom factor that limits the functional contribution based on how much of the disability is already accounted for by PTA (Table 9).

**Table 9:** Explanation of formula components PTA: pure-tone audiometry, ANPDQ: Auditory Neuropathy Perceived Disability Questionnaire.

Component	Description
PTA-based %	The hearing disability percentage derived using conventional method
ANPDQ %	(Score obtained / maximum score) × 100
K	A scaling constant (recommended k = 1.0 for initial application), allowing for future tuning based on empirical validation
(1 - PTA-based % / 100)	A headroom factor that proportionally scales down the contribution of ANPDQ as PTA-based disability increases, preventing overestimation.

Justification for the headroom

Double counting is avoided in the calculation, keeping the total disability within 100%. The ANPDQ contributes more when PTA underestimates disability (as in ANSD) and less when PTA already reflects substantial impairment. When PTA is low, the ANPDQ has greater influence, correcting the underestimation, and when the PTA is high, the ANPDQ contribution appropriately diminishes. This creates a balanced and bounded estimate that reflects real-world challenges more accurately. An illustrative example is given in Table 10. This weighting strategy is analogous to models used in other domains, such as visual disability [29] and the American Medical Association’s Guides to the Evaluation of Permanent Impairment [30], which combine impairments across systems using bounded additive methods. The formula should currently be considered exploratory. Future research could employ regression modelling to optimize weightings based on patient-reported outcomes, communication performance, and quality-of-life indicators, ultimately refining the tool for use in certification or benefit determination processes.

**Table 10:** Illustrative examples

Case	PTA %	ANPDQ score	ANPDQ %	Headroom factor	ANPDQ contribution	Final disability %
A	18%	82/164	50%	0.82	41%	59%
B	40%	82/164	50%	0.60	30%	70%
C	70%	82/164	50%	0.30	15%	85%
D	0%	82/164	50%	1.00	50%	50%

In summary, the PCA results validate the ANPDQ as a psychometrically sound, conceptually grounded, and clinically useful instrument for assessing disability in individuals with ANSD. The tool’s alignment with the ICF framework enhances its adaptability across health systems and cultures. It moves beyond the limitations of audiometric measures by foregrounding the lived experience of the disorder capturing communication breakdowns, psychosocial impact, listening effort, and participation restrictions. Given the limitations of current assessment frameworks, integrating questionnaire-based tools like the ANPDQ into clinical and disability evaluation systems is not just recommended but necessary to ensure accurate, inclusive, and compassionate care for individuals with ANSD.

The ANPDQ was developed and validated from a single tertiary care center, which may limit the generalizability of the findings to other settings or populations. Although participants were functionally literate in English, some degree of language-related bias cannot be ruled out. Additionally, it has not yet undergone translation or cross-cultural adaptation, which is important for its use in multilingual and international contexts. The decision not to compare the ANPDQ with existing hearing disability or auditory processing questionnaires was intentional, given the condition-specific focus of this tool. Future studies should explore these aspects, including cross-linguistic validation, comparisons with broader auditory disability measures, and longitudinal sensitivity to change in clinical or rehabilitative interventions.

## Conclusions

The findings of this study underscore the urgent need to rethink disability assessment in individuals with ANSD. The ANPDQ captures the nuanced and multifactorial nature of the disability experienced by this population, particularly in domains such as communication in noise, listening effort, and psychosocial impact, which are poorly reflected by traditional audiometric measures. By aligning closely with the lived experiences of individuals with ANSD, the ANPDQ offers a condition-specific, clinically relevant, and policy-relevant tool. Its integration into existing disability evaluation frameworks could lead to more accurate and equitable certification processes, thereby ensuring that individuals with ANSD receive the recognition and support they deserve. Future cross-cultural validations and longitudinal studies may further enhance its applicability in global contexts.

## Appendices

Supplementary material 1

**Table 11:** Domains for focus group discussion

Ease of communication	Relevance	Clarity	Simplicity
Do you have difficulty conversing with your family members?			
Do you easily understand the speech of an unfamiliar person?			
Do you have difficulty understanding speech in one-to-one conversation?			
Do you ask for repetition during a conversation?			
Do you take a longer time to respond to a question?			
Do you hesitate to initiate a conversation?			
Do you easily converse over the telephone?			
Do you have difficulty in understanding speech without looking at the speaker?			
Do you easily follow a conversation even without knowing the context?			
Do you miss out on a portion of the speech in the conversation?			
Do you easily understand speech when spoken louder?			
Comments:			

Supplementary material 2 

**Table 12:** Auditory Neuropathy Perceived Disability Questionnaire (ANPDQ)

Domains and questions
Ease of communication
1. Do you have difficulty conversing with your family members?
2. Do you easily understand the speech of an unfamiliar person?
3. Do you have difficulty understanding the speech of others in one-to-one conversation?
4. Do you ask for repetition during conversation?
5. Do you take longer to respond to a question?
6. Do you hesitate to initiate conversation?
7. Do you easily converse over the telephone?
8. Do you have difficulty understanding speech without looking at the speaker?
9. Do you easily follow conversation even without knowing the context?
10. Do you miss out on a portion of the speech in the conversation?
Communication in the background noise and reverberation
11. Do you easily carry out conversation in a crowded place (e.g., market)?
12. Do you have difficulty listening to TV in the background noise (e.g., family members talking, AC/fan running)?
13. Do you have difficulty carrying out a telephonic conversation in the background noise (e.g., busy street/traffic)?
14. Do you easily understand speech in a classroom/theatre/open hall/lecture room?
15. Do you misunderstand parts of the conversation in the background noise?
16. Do you have difficulty carrying out conversation with your fellow passengers during travel (e.g., auto, bus, train, flight)?
17. Do you easily carry out one-to-one conversation in a quiet place?
Spatial hearing
18. Do you have difficulty judging the direction of the vehicle by listening?
19. Do you have difficulty determining the source of the sound by listening?
20. Do you have difficulty judging the distance of a sound source by listening?
21. Do you have difficulty recognizing who is talking in a crowd?
Quality of heard speech and non-speech sounds
22. Do you find it easy to recognize people by their voices?
23. Do you feel clarity of speech is poor over the telephone?
24. Do you feel environmental sounds sound natural to you?
25. Do you have difficulty perceiving the emotion/intention of the talker by the voice?
26. Do you find clarity of speech is better when spoken louder?
27. Do you have difficulty differentiating between speech sounds and environmental sounds?
28. Do you have difficulty appreciating the quality of music?
29. Do you have difficulty identifying the non-speech sounds?
Activities and participation
30. Do you have difficulty attending school/college/work due to hearing issues?
31. Do you have difficulty attending social gatherings?
32. Do you find it easy to socialize with others?
33. Do you have difficulty involving yourself in recreational and leisure activities?
34. Do you have difficulty maintaining friendly relationships?
35. Do you find it difficult to acquire new skills due to hearing issues?
Psychological impact
36. Do you feel sad because of hearing difficulty?
37. Have you lost interest in other people and activities?
38. Do you feel frustrated because of hearing difficulty?
39. Do you have a fear of failure because of hearing difficulty?
40. Do you feel worthless because of hearing difficulty?
41. Do you feel you have adequate sleep?
42. Do you feel your eating pattern is disturbed?
43. Do you feel anxious because of hearing difficulty?
44. Do you feel irritable because of hearing difficulty?
45. Do you feel uncertain about your future because of hearing difficulty?
46. Do you feel neglected because of hearing difficulty?
47. Do you have anger issues because of hearing difficulty?
48. Do you feel emotionally drained due to hearing difficulty?
49. Do you have memory issues?
Sensory, motor, and balance
50. Do you experience giddiness?
51. Do you have difficulty maintaining balance in dark/eyes closed conditions?
52. Do you easily climb stairs?
53. Do you hear ringing sound in your ears?
54. Do you have difficulty standing or walking?
55. Do you find it easy to perform everyday tasks independently?
Listening effort and fatigue
56. Do you have to concentrate more during conversation?
57. Do you get fatigued easily due to listening?
58. Do you feel exhausted after a conversation?
59. Do you think you can listen effortlessly over the telephone?
Overall disability

## References

[1] Starr A, Picton TW, Sininger Y, Hood LJ, Berlin CI (1996). Auditory neuropathy. Brain.

[2] Rance G (2005). Auditory neuropathy/dys-synchrony and its perceptual consequences. Trends Amplif.

[3] Rance G, Barker E, Mok M, Dowell R, Rincon A, Garratt R (2007). Speech perception in noise for children with auditory neuropathy/dys-synchrony type hearing loss. Ear Hear.

[4] Penido RC, Isaac ML (2013). Prevalence of auditory neuropathy spectrum disorder in an auditory health care service. Braz J Otorhinolaryngol.

[5] Midgley E (2013). The prevalence of auditory neuropathy spectrum disorder in neonates referred from the newborn hearing screening programme in Avon (Greater Bristol area). Cochlear Implants International.

[6] Kumar UA, Jayaram MM (2006). Prevalence and audiological characteristics in individuals with auditory neuropathy/auditory dys-synchrony. Int J Audiol.

[7] Narne VK, Prabhu P, Chandan HS, Deepthi M (198). Audiological profiling of 198 individuals with auditory neuropathy spectrum disorder. Hearing, Balance, and Communication.

[8] Muthukumar R, Jaya V, Vignesh SS, Thenmozhi K (2023). Prevalence and auditory characteristics of auditory neuropathy spectrum disorder in adult population with sensory neural hearing loss: a hospital based study in South India. Indian J Otolaryngol Head Neck Surg.

[9] Vignesh SS, Jaya V, Muraleedharan A (2016). Prevalence and audiological characteristics of auditory neuropathy spectrum disorder in pediatric population: a retrospective study. Indian J Otolaryngol Head Neck Surg.

[10] Deltenre P, Mansbach AL, Bozet C, Christiaens F, Barthelemy P, Paulissen D, Renglet T (1999). Auditory neuropathy with preserved cochlear microphonics and secondary loss of otoacoustic emissions. Audiology.

[11] Rance G, Beer DE, Cone-Wesson B (1999). Clinical findings for a group of infants and young children with auditory neuropathy. Ear and Hearing.

[12] Kumar AU, Jayaram M (2005). Auditory processing in individuals with auditory neuropathy. BBF.

[13] Yuvaraj P, Jayaram M (2016). Audiological profile of adult persons with auditory neuropathy spectrum disorders. J Audiol Otol.

[14] De Siati RD, Rosenzweig F, Gersdorff G, Gregoire A, Rombaux P, Deggouj N (2020). Auditory neuropathy spectrum disorders: from diagnosis to treatment: literature review and case reports. J Clin Med.

[15] Zeng FG, Oba S, Garde S, Sininger Y, Starr A (1999). Temporal and speech processing deficits in auditory neuropathy. Neuroreport.

[16] Kraus N, Bradlow AR, Cheatham MA (2000). Consequences of neural asynchrony: a case of auditory neuropathy. J Assoc Res Otolaryngol.

[17] Zeng FG, Kong YY, Michalewski HJ, Starr A (2005). Perceptual consequences of disrupted auditory nerve activity. J Neurophysiol.

[18] Kim H, Park JY, Choung YH, Jang JH, Ko J (2021). Predicting speech discrimination scores from pure-tone thresholds-a machine learning-based approach using data from 12,697 subjects. PLOS One.

[19] Shetty HN, Mathai JP, Uppunda AK (2017). Disability certificate for individuals with hearing impairment: Time to rethink. Indian Journal of Otology.

[20] World Health Organization. (2001 (2001). World Health Organization. International classification of functioning, disability and health (ICF). https://www.who.int/standards/classifications/international-classification-of-functioning-disability-and-health.

[21] Starr A, Sininger YS, Pratt H (2000). The varieties of auditory neuropathy. J Basic Clin Physiol Pharmacol.

[22] Hogarty KY, Hines CV, Kromrey JD, Ferron Ferron, JM JM, Mumford KR (2005). The quality of factor solutions in exploratory factor analysis: the influence of sample size, communality, and overdetermination. Educ Psychol Meas.

[23] Hutcheson G, Sofroniou N The Multivariate Social Scientist. Introductory Statistics Using Generalized Linear Models. https://uk.sagepub.com/en-gb/eur/the-multivariate-social-scientist/book205684.

[24] Zeng Zeng, F. G., & Liu, S. (2006 (2006). Speech perception in individuals with auditory neuropathy. J Speech Lang Hear Res.

[25] Gatehouse S, Noble W (2004). The Speech, Spatial and Qualities of Hearing Scale (SSQ). Int J Audiol.

[26] Ministry of Social Justice and Empowerment. (2023 (2023). Ministry of Social Justice and Empowerment. Rights of persons with disabilities (amendment) rules. Rights of Persons with Disabilities (Amendment) Rules.

[27] MS V, Madoure A, Raja K (2024). Criteria for determining hearing disability: a narrative review of global perspective. Indian J Otolaryngol Head Neck Surg.

[28] Park MS, Kang KJ, Jang SJ, Lee JY, Chang SJ (2018). Evaluating test-retest reliability in patient-reported outcome measures for older people: a systematic review. Int J Nurs Stud.

[29] World Health Organization. (2018 (2018). World Health Organization. International Classification of Diseases for Mortality and Morbidity Statistics (11th Revision). https://icd.who.int/en/.

[30] American Medical Association (2008). AMA Guides® to the Evaluation of Permanent Impairment, Sixth Edition. https://ama-guides.ama-assn.org/display/book/9781640162822/9781640162822.xml.

